# Thrombus or tumor? A case report of a rare sarcoma entity: intimal sarcoma of the pulmonary arteries

**DOI:** 10.1007/s11033-024-09467-9

**Published:** 2024-04-24

**Authors:** A. Dörr, A. Flörcken, L. Bullinger, D. Capper, A. Von Deimling, D. Kaul, S. Märdian, C. Starck, D. Horst, M.P. Dragomir, F.M. Schäfer, A. Jarosch

**Affiliations:** 1 Department of Hematology, Oncology, and Tumor Immunology, Charité - Universitätsmedizin Berlin, Corporate member of Freie Universität Berlin, Humboldt-Universität zu Berlin and Berlin Institute of Health, Berlin, Germany; 2 German Cancer Consortium (DKTK), Partner site Berlin, German Cancer Research Center (DKFZ), Heidelberg, Germany; 3 Department of Neuropathology, Charité - Universitätsmedizin Berlin, corporate member of Freie Universität Berlin, Humboldt-Universität zu Berlin and Berlin Institute of Health, Berlin, Germany; 4 Department of Neuropathology, and CCU Neuropathology, University Hospital Heidelberg, DKFZ, Heidelberg, Germany; 5 Department of Pathology, Charité - Universitätsmedizin Berlin, Corporate member of Freie Universität Berlin, Humboldt-Universität zu Berlin, Berlin, Germany; 6 Department of Radiation Oncology, Charité - Universitätsmedizin Berlin, Corporate member of Freie Universität Berlin and Humboldt and Universität zu Berlin and Berlin Institute of Health, Berlin, Germany; 7 Center for Musculoskeletal Surgery, Charité - Universitätsmedizin Berlin, Corporate member of Freie Universität Berlin and Humboldt Universität zu Berlin and Berlin Institute of Health, Berlin, Germany; 8 Department of Cardiothoracic and Vascular Surgery, Deutsches Herzzentrum der Charité (DHZC), Berlin, Germany; 9 Berlin Institute of Health (BIH), Berlin, Germany; 10 Institute for Radiology, Charité - Universitätsmedizin Berlin, Universität zu Berlin, Corporate member of Freie Universität Berlin and Humboldt, Berlin, Germany

**Keywords:** Intimal sarcoma, Rare entity, Pulmonary arteries, DNA methylation

## Abstract

**Background:**

Tumor embolism is a very rare primary manifestation of cancers and the diagnosis is challenging, especially if located in the pulmonary arteries, where it can mimic nonmalignant pulmonary embolism. Intimal sarcoma is one of the least commonly reported primary tumors of vessels with only a few cases reported worldwide. A typical location of this malignancy is the pulmonary artery. Herein, we present a case report of an intimal sarcoma with primary manifestation in the pulmonary arteries.

**Case summary:**

A 53-year-old male initially presented with dyspnea. On imaging, a pulmonary artery embolism was detected and was followed by thrombectomy of the right ventricular outflow tract, main pulmonary artery trunk, and right pulmonary artery after ineffective lysis therapy. Complementary imaging of the chest and abdomen including a PET-CT scan demonstrated no evidence of a primary tumor. Subsequent pathology assessment suggested an intimal sarcoma further confirmed by DNA methylation based molecular analysis. We initiated adjuvant chemotherapy with doxorubicin. Four months after the completion of adjuvant therapy a follow-up scan revealed a local recurrence without distant metastases.

**Discussion:**

Primary pulmonary artery intimal sarcoma (PAS) is an exceedingly rare entity and pathological diagnosis remains challenging. Therefore, the detection of entity-specific molecular alterations is a supporting argument in the diagnostic spectrum. Complete surgical resection is the prognostically most important treatment for intimal cardiac sarcomas. Despite adjuvant chemotherapy, the prognosis of cardiac sarcomas remains very poor. This case of a PAS highlights the difficulty in establishing a diagnosis and the aggressive natural course of the disease.

**Conclusion:**

In case of atypical presentation of a pulmonary embolism, a tumor originating from the great vessels should be considered. Molecular pathology techniques support in establishing a reliable diagnosis.

**Supplementary Information:**

The online version contains supplementary material available at 10.1007/s11033-024-09467-9.

## Introduction

Tumors originating from blood vessels may arise from endothelial cells (hemangioma, lymphangioma, hemangioendothelioma, angiosarcoma) or cells supporting or surrounding blood vessels (glomus tumor, myopericytoma, angioleiomyoma). Most of these tumors arise in the soft tissues or within organs. Primary tumors of the large vessels (e.g. aorta, inferior vena cava) are very rare and are usually connective tissue sarcomas [1]. First reported in 1923 by Mandelstamm, primary pulmonary artery sarcoma (PAS) is an extremely rare malignant neoplasm [2]. This unique sarcoma entity mimics pulmonary embolism both clinically and on imaging, leading to diagnostic difficulties and significant therapeutic delay [3]. Due to the rarity of PAS most of our knowledge derives from case reports and small case series [4], [5], [6], [7]. The laboratory findings, clinical symptoms, and radiologic imaging are non-specific which results in diagnostic challenges. Moreover, there is no standard treatment for patients with PAS.

### Case report

A 53-year-old male was referred to our hospital with dyspnea and chest pain following a Covid-19 infection, being suspected of post-COVID lung disease. He had no significant medical history. The family history was positive for cancer with the patient’s father suffering from head and neck squamous cell carcinoma, an uncle suffering from colorectal cancer, and a cousin from lymphoma. The patient had no occupational exposure to hazardous material, and he denied recent travels. The Patient identified as a current smoker. The laboratory tests revealed no abnormalities. Both the blood count and the clinical chemistry were unremarkable.

**Fig. 1:**
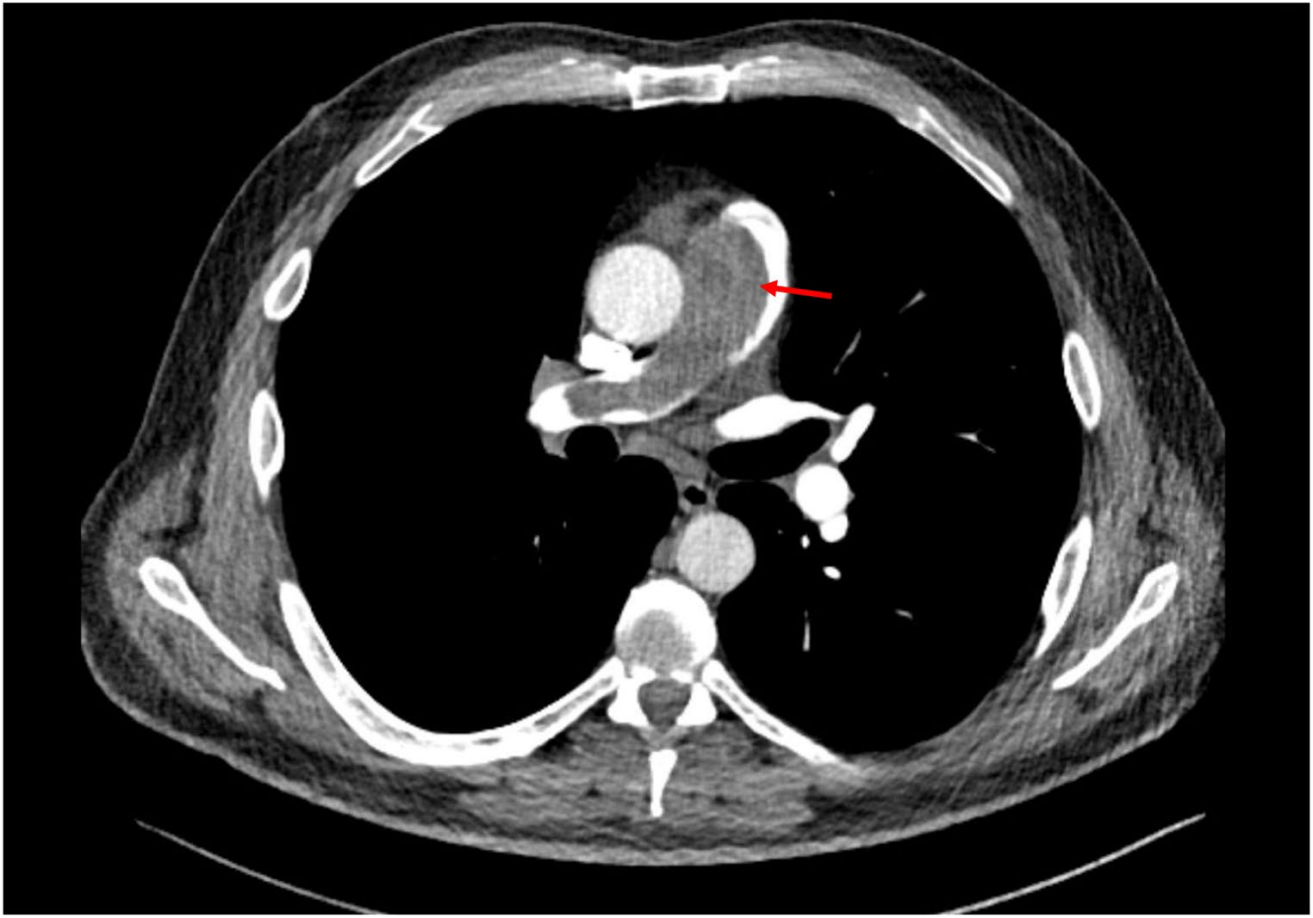
CT-angiography showing a bilateral pulmonary artery embolism with large central thrombus (red arrow) in the pulmonary trunk

Initial imaging included echocardiography and computed tomography (CT). The echocardiography showed acute right heart strain with D-shape and reduced RVEF. A CT angiography revealed pulmonary artery embolism with a large central mass, suspected to be of thrombotic origin, in the pulmonary trunk with subtotal occlusion and extension into the right pulmonary artery (Fig. 1).

Significant coronary artery disease was ruled out by cardiac catheterization. Lysis therapy was ineffective, without significant thrombus regression. The patient underwent thrombectomy of the right ventricular outflow tract, as well as the main pulmonary artery trunk, and the right pulmonary artery.

**Fig. 2:**
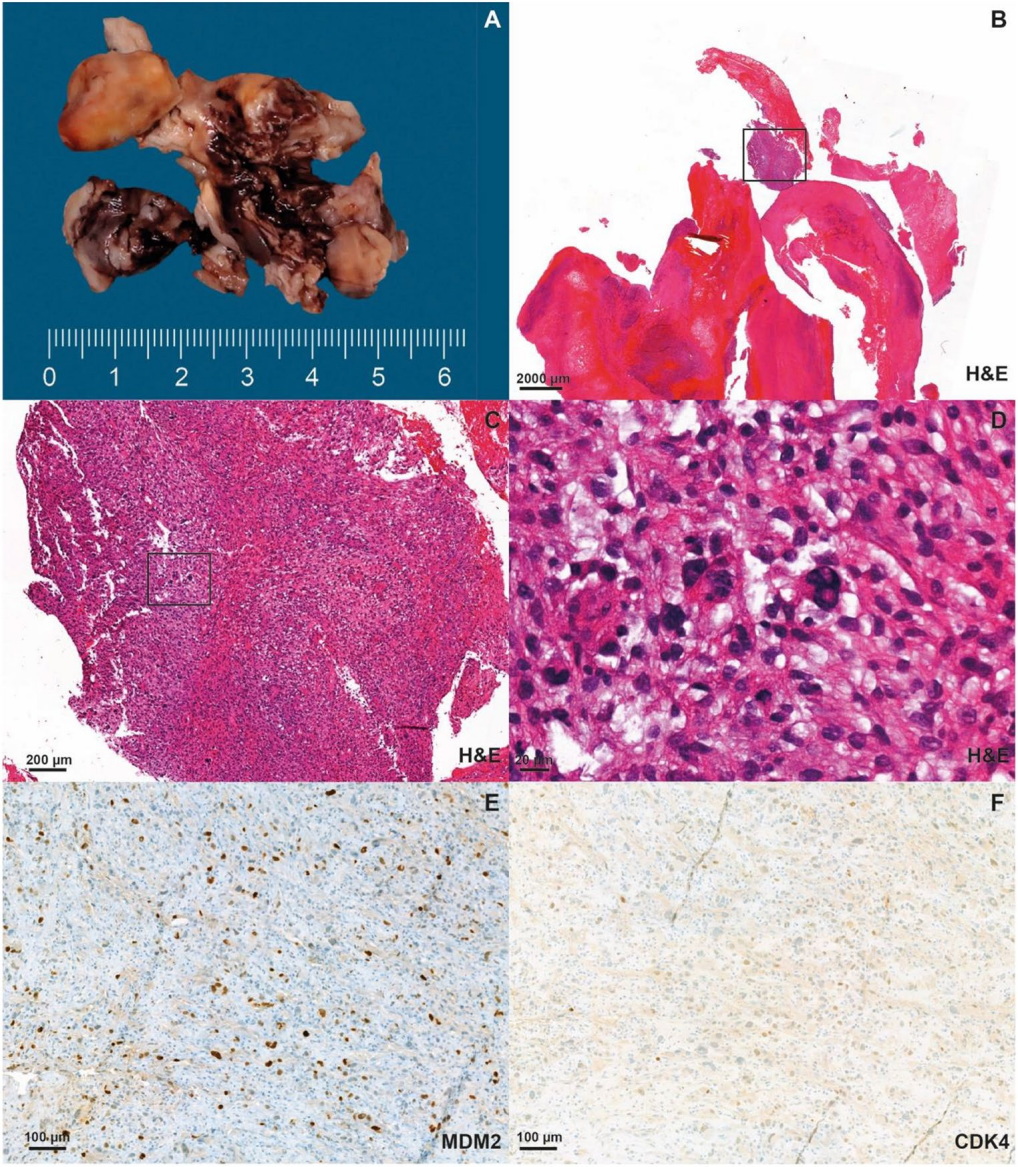
Representative macroscopic image of the tumor thrombus prior to gross processing (**A**). Low power filed microscopy image of the tumor thrombus, H&E staining, (**B**), medium power field image of the hypercellular areas of the specimen, H&E staining (**C**), high power field showing cellular pleomorphism and increased mitotic activity (**D**), immunohistochemistry showing strong nuclear expression of MDM2 (**E**) and weak nuclear expression of CDK4 (**F**). A scale bar for each image is located in the lower left corner of each panel

Pathologic examination of the thrombus (Fig. 2A) revealed a malignant embolus composed of older thrombotic material and a tumor with part-clear-cell and part-spindle-cell morphology (Fig. 2B-D). Initially, an underlying renal cell carcinoma with sarcomatoid growth was suspected due to the clear cell morphology. However, an MRI of the abdomen showed no evidence of a primary kidney tumor. The physical examination revealed no suspicious mass, particularly not on the extremities.

Histologically, the embolus contained portions of a poorly differentiated mesenchymal tumor compatible with a high-grade sarcoma (Fig. 2D). For further characterization, additional cyclin-dependent kinase 4 (CDK4) and Murine double minute 2 (MDM2) immunohistochemistry was performed. MDM2 showed a strong nuclear expression (Fig. 2E) whereas CDK4 showed only weak expression in some tumor cell nuclei (Fig. 2F).

**Fig. 3:**
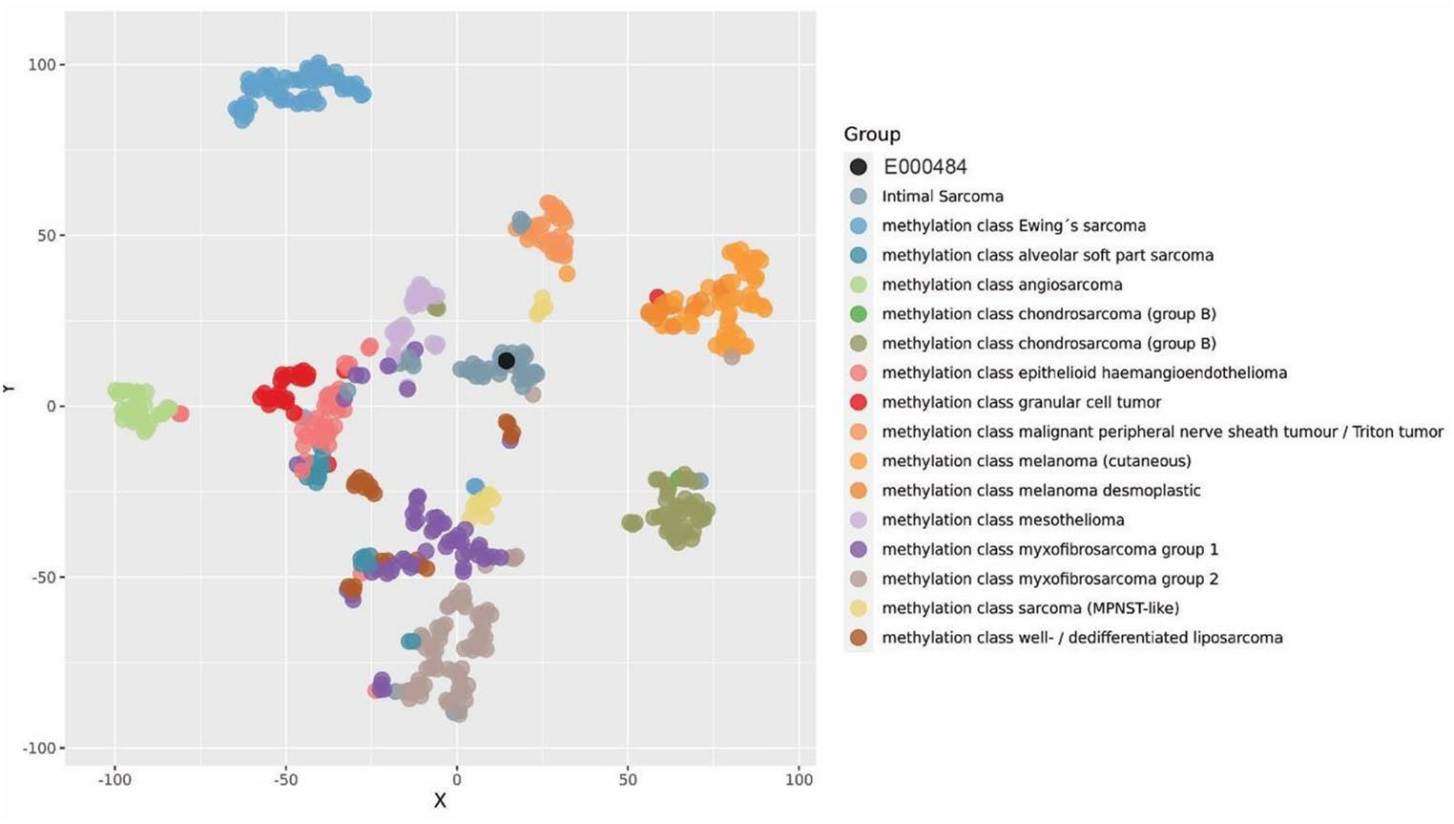
t-SNE analysis with an ad hoc reference set of soft tissue and bone tumors that also included 26 intimal sarcomas that were specifically assembled for this case. The t-SNE analysis revealed clear clustering of our case (black dot) with the other intimal sarcomas of the cohort (gray dots)

Further immunohistochemical staining against pan-cytokeratin, CD31, CD34, desmin, smooth muscle actin (SMA), and S100 showed no positive expression. Subsequently, in-situ hybridization was performed. Here, there was evidence of *MDM2* amplification seen by positive *MDM2*-Amp-FISH analysis. Considering the morphology and the *MDM2* amplification, we initially suspected a pulmonary metastasis of a dedifferentiated liposarcoma from a different localization. A PET-CT scan was performed and showed no clear evidence of a primary tumor making a metastatic dedifferentiated liposarcoma implausible. For further differential diagnosis, tumor DNA was extracted, and genome-wide DNA methylation was performed. The genome-wide DNA methylation profile was introduced into the Sarcoma Classifier [8] and was labeled as a “no match”, thus not meeting the threshold for any of the classifier’s sarcoma subtypes. This would rather argue against a type of sarcoma that is included in the reference set of the Sarcoma Classifier (among others dedifferentiated liposarcoma). Based on localization, morphology, and *MDM2* amplification, intimal sarcoma was an additional suspected diagnosis. Intimal sarcomas are not included in the published reference set of the Sarcoma Classifier and therefore no predicted methylation class exists for these tumors in the classifier. Therefore, we created an ad hoc reference set that also included 26 intimal sarcomas that were specifically assembled for this case. We used the DNA methylation profile of our case and of the ad hoc reference set samples and performed a t-distributed stochastic neighbor embedding (t-SNE) analysis. Here, the tumor showed clear clustering with the intimal sarcoma sample group (Fig. 3).

**Fig. 4:**
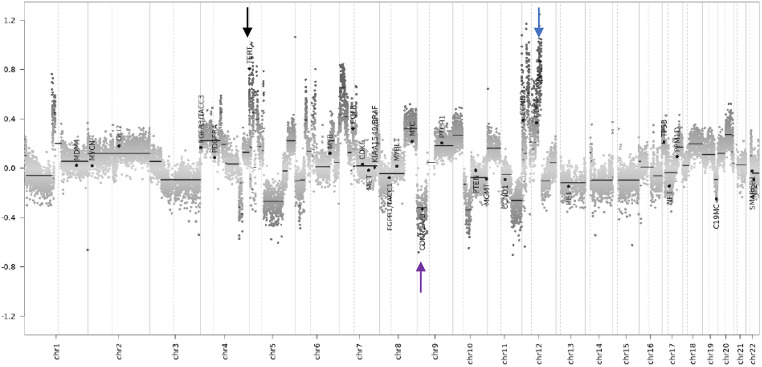
Copy number profile of the case sample showing MDM2 (blue arrow) and TERT (black arrow) amplification and CDKN2A/B deletion (purple arrow), alterations that are well compatible with the diagnosis of intimal sarcoma

We also used the DNA methylation data to perform copy number analysis. The copy number alterations further confirmed the *MDM2* amplification but also revealed *TERT* amplification as well as *CDKN2A/B* deletion (Fig. 4) that are compatible with the diagnosis of intimal sarcoma [9].

Thus, the DNA methylation-based Sarcoma Classifier argued against a dedifferentiated liposarcoma, and the clustering of our sample in the t-SNE analysis with the intimal sarcoma group and the copy number alterations supported the diagnosis of an intimal sarcoma. In conclusion, the histomorphology and immunohistochemical expression profile in combination with the DNA-methylation profiling results plus the radiological images lead to the diagnosis of an intimal sarcoma with origin in the pulmonary arteries.

Since tumor thrombectomy represents at least an R1, probably an R2 surgery, we initiated adjuvant systemic therapy with doxorubicin. In total, the patient completed five cycles of chemotherapy. A sixth cycle was planned but was not administered due to very poor tolerability. The patient experienced severe nausea and vomiting as well as recurrent oral mucositis. The treatment was also complicated by fatigue and weight loss. Following the fifth cycle of doxorubicin the patient developed a fever of unknown origin which required antibiotic treatment.

The first posttreatment CT with contrast demonstrated stable disease with constant, approximately circular wall thickening of the left main pulmonary trunk. The CT morphology was not able to distinguish tumor portions of the intimal sarcoma from adjacent thrombi.

**Fig. 5:**
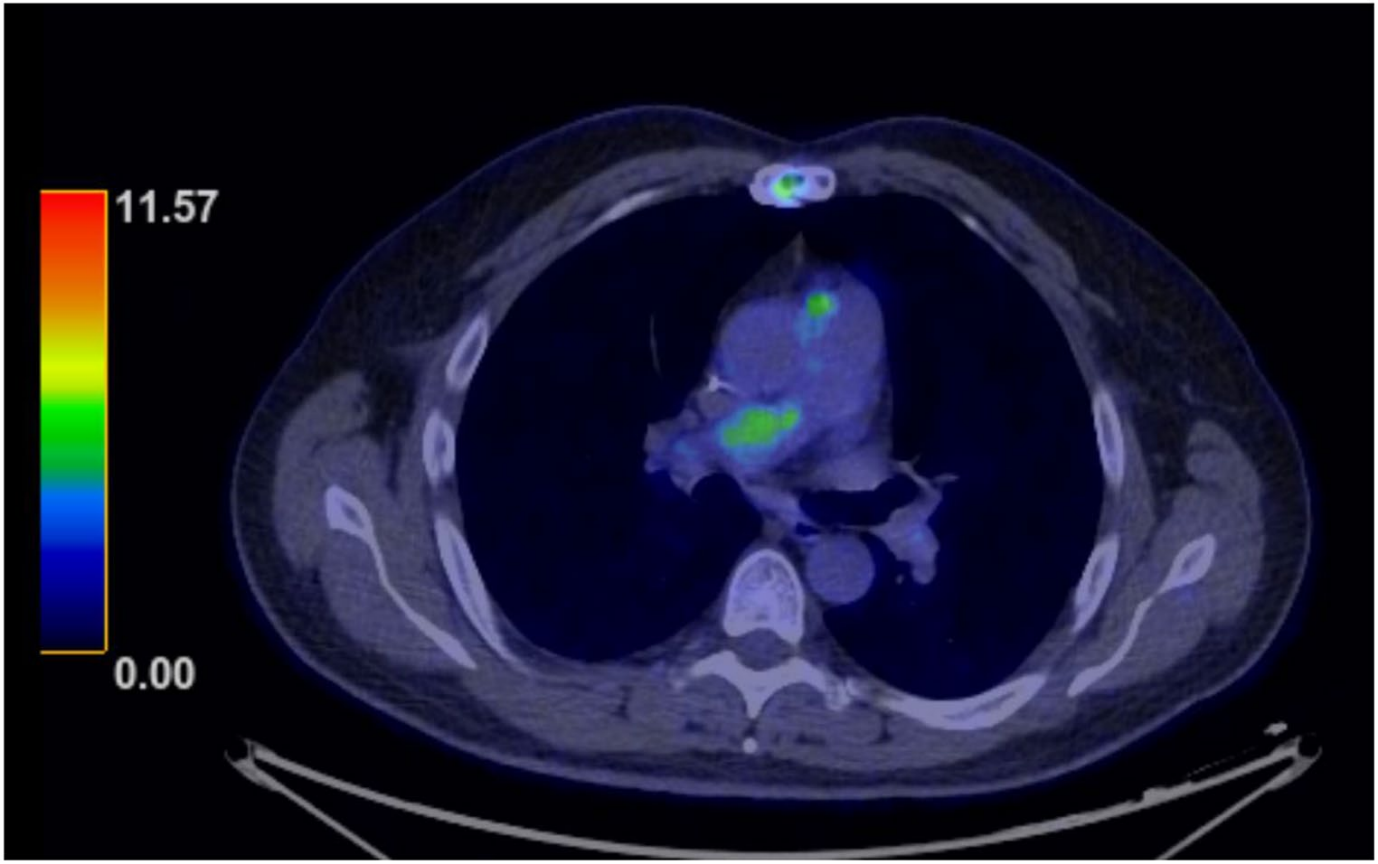
PET-CT showing an axial view of the recurrent, metabolically active mass in the pulmonary trunk

Four months after the completion of adjuvant therapy and 8 months after the tumor thrombectomy, we detected local progression/recurrence in the pulmonary arterial vasculature and in the left main pulmonary trunk. An additional PET-CT indicated a trifocal recurrence in the region of the truncus pulmonalis, in the area of the right pulmonary trunk, and in the region of the left pulmonary trunk (Fig. 5). There was no indication of distant metastases. The patient refused further chemotherapy due to the toxicity experienced with doxorubicin therapy. As a result, we initiated a systemic therapy with the multikinase inhibitor pazopanib. Just two months later we detected rapid and significant progression on another CT scan and thereupon initiated chemotherapy with ifosfamide, which induced a partial remission. If the remission is either stable or we will see further reduction in tumor size, we intend to evaluate the patient for enrollment in a clinical trial testing an Mdm2 inhibitor.

## Discussion

Here we present a case of high-grade intimal sarcoma with origin in the pulmonary arteries. Our patient presented with nonspecific symptoms related to right heart failure. Imaging results were not conclusive. The diagnostic challenge with an intravascular mass is to differentiate between a thrombus, a tumor thrombus, or a tumor mass. A PET-CT can contribute to the diagnosis with a high metabolic uptake suggesting a malignancy. A nonmalignant lesion such as pulmonary thromboembolism would show a low uptake. In our case, the tumor was suspicious of pulmonary artery embolism leading to acute right heart failure. Therefore, a tumor-thrombectomy was performed. Surgical intervention is considered the mainstay treatment to eliminate pulmonary artery obstruction and associated pulmonary hypertension and to palliate symptoms [10]. Pathology and further immunohistochemical and molecular genetic analyses provided the final diagnosis. To determine the optimal treatment, it is essential to develop a definitive diagnosis as treatment and prognosis vary drastically for malignant lesions such as pulmonary artery intimal sarcoma or metastases from other cancers or benign pulmonary thromboembolism.

Intimal sarcomas are undifferentiated mesenchymal tumors. They typically arise from the inner lining of the large vessels of the heart or in the left atrium. Differentiation into various tissue types, including cartilage and bone, as well as angiosarcoma and rhabdomyosarcoma have been reported [11]. In our case, we found no evidence of differentiation into other sarcoma entities in either radiological images or histology.

Molecular studies revealed a high frequency of *MDM2* overexpression and amplifications in intimal sarcomas. *MDM2* alterations are oftentimes accompanied by co-amplifications of *CDK4*, *PDGFRA*, and *TERT* [9], [12]. In our case, we found *MDM2* overexpression but only weak staining for *CDK4*. Despite these morphological and molecular assays, differential diagnostics of sarcoma arising in the vessels remains challenging. Genome-wide DNA methylation arrays with machine-learning classifiers provide a powerful tool for robust molecular tumor classification. An important limitation of this approach is the difficulty in assembling the necessary samples for a rare tumor as part of the reference set of the classifier. The Sarcoma Classifier does not include intimal sarcomas and therefore cannot diagnose this entity. Koelsche et al. characterized a cohort of 26 intimal sarcomas and 9 undifferentiated pleomorphic sarcomas of the left atrium using genome-wide DNA methylation profiling to determine the copy number profile of this entity and its clustering with other sarcoma types [8]. The intimal sarcoma methylation signature was distinct from potential histologic and molecular mimics. Herein, we performed a t-SNE analysis of our case sample with the reference set assembled by Koelsche and colleagues for the characterization of intimal sarcomas. Our sample clustered with the intimal sarcoma group, supporting the diagnosis of intimal sarcoma. Our case report highlights the importance of international collaborations to achieve the necessary sample size to expand globally available DNA methylation classifiers, such as the Sarcoma Classifier, for very rare entities, such as intimal sarcomas. Additionally, the copy number variation profile revealed *MDM2* and *TERT* amplification, well in line with the observations by Koelsche et al. and Roszik et al. [13].

As in sarcomas originating from different sites, e.g., the extremities, the trunk wall, or the retroperitoneum, complete surgical resection is the optimal treatment of intimal sarcomas. Nonetheless, in most cases, surgery is noncurative as it is difficult to achieve clear (R0) margins. If feasible, adjuvant chemotherapy or radiation should follow surgery to achieve better disease control and to improve the prognosis [14]. Retrospective data on the activity of systemic therapies in intimal sarcoma are limited and no prospective studies have been conducted. Although adjuvant anthracycline-based chemotherapy leads to a promising response rate (RR) of 38% the prognosis remains poor. A study of 66 patients receiving anthracyclines reported recurrence-free survival (RFS) of 14.6 months in patients with localized disease and progression-free survival (PFS) of 7.7 months in patients with advanced disease [15]. In our case, progression after anthracycline therapy occurred 8 months after the initial surgery, according to the previously described PFS. For second-line therapy we chose pazopanib, mainly because of the better tolerable side effects. As pazopanib is known to have limited activity in achieving complete responses, we are aiming for disease stabilization [11].

Despite multiple and multidisciplinary treatment options, the prognosis for pulmonary artery intimal sarcoma remains dismal due to the difficulty in achieving complete surgical resection. In the metastatic setting, chemotherapy and radiation therapy are the primary treatment options and should be considered if surgery is unfeasible.

As also detected in our patient, *MDM2* amplification is observed in > 70% of intimal sarcomas [16], and targeted therapy against *MDM2* has shown some promise in small clinical studies [17]. Therefore, inhibition of *MDM2* might be a feasible strategy in this exceedingly rare and highly aggressive entity with limited treatment options.

## Conclusion

PAS is an extremely rare cancer entity, which is often misdiagnosed as pulmonary embolism. PAS should be considered in atypical clinical presentations with inadequate response to lysis therapy. A high level of pathology expertise, including the use of molecular pathology techniques such as genome-wide DNA methylation, is required to establish an accurate and reliable diagnosis.

## Electronic supplementary material

Below is the link to the electronic supplementary material.

Supplementary Material 1

## Data Availability

The reference set for DNA methylation profile analysis is available from the authors upon request.
