# Supplementary material for: Thrombus or tumor? A case report of a rare sarcoma entity: intimal sarcoma of the pulmonary arteries
Source: Mol Biol Rep. 2024 Apr 24;51(1):568. doi: 10.1007/s11033-024-09467-9 (PMC11043182; doi:10.1007/s11033-024-09467-9)
Supplement: Supplementary file 1 — Supplementary Material 1 [file 11033_2024_9467_MOESM1_ESM.pdf]

CARE case report guidelines

CARE Checklist of information to include when writing a case report

(cc) BY-NC-ND

Topic	Item	Checklist item description	Reported on Line
Title	1	The diagnosis or intervention of primary focus followed by the words "case report"	1-2
Key Words	2	2 to 5 key words that identify diagnoses or interventions in this case report, including "case report"	50
Abstract (no references)	3a	Introduction: What is unique about this case and what does it add to the scientific literature?	59-64
	3b	Main symptoms and/or important clinical findings	66 - 82
	3c	The main diagnoses, therapeutic interventions, and outcomes	83-152
	3d	Conclusion—What is the main "take-away" lesson(s) from this case?	181-186
Introduction	4	One or two paragraphs summarizing why this case is unique (**may include references**)	105-134
Patient Information	5a	De-identified patient specific information.	n/a
	5b	Primary concerns and symptoms of the patient.	66-67
	5c	Medical, family, and psycho-social history including relevant genetic information	68-71
	5d	Relevant past interventions with outcomes	n/a
Clinical Findings	6	Describe significant physical examination (PE) and important clinical findings.	71-80, 87-88
Timeline	7	Historical and current information from this episode of care organized as a timeline	n/a
Diagnostic Assessment	8a	Diagnostic testing (such as PE, laboratory testing, imaging, surveys).	71-81
	8b	Diagnostic challenges (such as access to testing, financial, or cultural)	n/a
	8c	Diagnosis (including other diagnoses considered)	84-134
	8d	Prognosis (such as staging in oncology) where applicable	151-152
Therapeutic Intervention	9a	Types of therapeutic intervention (such as pharmacologic, surgical, preventive, self-care)	136-151
	9b	Administration of therapeutic intervention (such as dosage, strength, duration)	n/a
	9c	Changes in therapeutic intervention (with rationale)	145-152
Follow-up and Outcomes	10a	Clinician and patient-assessed outcomes (if available)	n/a
	10b	Important follow-up diagnostic and other test results	145-152
	10c	Intervention adherence and tolerability (How was this assessed?)	136-141
	10d	Adverse and unanticipated events	136-141
Discussion	11a	A scientific discussion of the strengths AND limitations associated with this case report	172-186
	11b	Discussion of the relevant medical literature **with references**.	178-197
	11c	The scientific rationale for any conclusions (including assessment of possible causes)	205-208
	11d	The primary "take-away" lessons of this case report (without references) in a one paragraph conclusion	201-204
Patient Perspective	12	The patient should share their perspective in one to two paragraphs on the treatment(s) they received	149-150
Informed Consent	13	Did the patient give informed consent? Please provide if requested	Yes ☒ No ☐
